# Two Lysine Sites That Can Be Malonylated Are Important for LuxS Regulatory Roles in *Bacillus velezensis*

**DOI:** 10.3390/microorganisms9061338

**Published:** 2021-06-21

**Authors:** Xianming Cao, Yulong Li, Jialu Fan, Yinjuan Zhao, Rainer Borriss, Ben Fan

**Affiliations:** 1 College of Forestry, Nanjing Forestry University, Nanjing 210037, China; CaoXianMing2018@gmail.com (X.C.); yulongli@njfu.edu.cn (Y.L.); zhaoyinjuan@njfu.edu.cn (Y.Z.); 2 College of Life Sciences, Nanjing Normal University, Nanjing 210046, China; 181202012@njnu.edu.cn; 3 Institut für Biologie, Humboldt Universität Berlin, 10115 Berlin, Germany; rainer.borriss@rz.hu-berlin.de; 4 Nord Reet UG, Marienstr. 27a, 17489 Greifswald, Germany

**Keywords:** LuxS, malonylation, AI-2, biofilm formation, swarming, sporulation, antibiotic production, in vitro enzymatic activity

## Abstract

S-ribosylhomocysteine lyase (LuxS) has been shown to regulate bacterial multicellular behaviors, typically biofilm formation. However, the mechanisms for the regulation are still mysterious. We previously identified a malonylation modification on K124 and K130 of the LuxS in the plant growth-promoting rhizobacterium *B.* *velezensis* (FZB42). In this work, we investigated the effects of the two malonylation sites on biofilm formation and other biological characteristics of FZB42. The results showed that the K124R mutation could severely impair biofilm formation, swarming, and sporulation but promote AI-2 production, suggesting inhibitory effects of high-level AI-2 on the features. All mutations (K124R, K124E, K130R, and K130E) suppressed FZB42 sporulation but increased its antibiotic production. The double mutations generally had a synergistic effect or at least equal to the effects of the single mutations. The mutation of K130 but not of K124 decreased the in vitro enzymatic activity of LuxS, corresponding to the conservation of K130 among various *Bacillus* LuxS proteins. From the results, we deduce that an alternative regulatory circuit may exist to compensate for the roles of LuxS upon its disruption. This study broadens the understanding of the biological function of LuxS in *bacilli* and underlines the importance of the two post-translational modification sites.

## 1. Introduction

Lysine malonylation is a type of dynamic and reversible protein post-translational modifications (PTM), which can affect many biological processes by regulating activities, localizations, and/or interactions of proteins involved in glycolysis and fatty acids metabolism pathways, malonic aciduria, type II diabetes, and other genetic diseases [1,2,3,4,5]. At present, studies on malonylation are mainly performed with eukaryotes, whereas only a few reports were focused on bacteria such as *B. velezensis* (FZB42) [1]. *B. velezensis* (FZB42) is a prototype of plant growth-promoting rhizobacteria (PGPR), which have been the subject of extensive investigations in the past decade due to their enormous economic values [6,7]. FZB42 can synthesize more than ten antimicrobial substances and form robust biofilm [6,8], both of which are inseparable features for its protective activities on plants, assisting plants in their defense against phytopathogens.

In the previous work, we analyzed the malonylome of FZB42, among which was the LuxS protein [1]. As a homodimer of hydrolase, LuxS catalyzes the break of the thioether bond of S-ribose homocysteine (SRH), producing homocysteine and 4,5-dihydroxy-2,3-pentanedione (DPD), which spontaneously cyclizes into active autoinducer-2 (AI-2) [9,10,11]. While SRH is a member of the activated methyl cycle (AMC), AI-2 serves as the signaling molecule in quorum sensing and is generally regarded as a universal language by bacteria to mediate their intra- and interspecific communication [12,13,14].

AI-2 is a furanosyl boronic acid diester [15,16]. Being secreted out of the cells after synthesis, AI can trigger quorum sensing when its extracellular concentration reaches the threshold [13]. AI-2 regulates various physiological functions of bacteria, such as bioluminescence, toxin production, and proteome modulation [14,17]. Two types of AI-2 receptors were firstly identified involving LuxP that was only found in *Vibrio* and LsrB and were detected in *E. coli* and *Salmonella* [18,19,20,21,22,23,24]. The latest research demonstrates that the proteins with a dCACHE (double calcium channels and chemotaxis receptors) domain, including KinD in *B. subtilis*, serve as a novel type of AI-2 receptors [25].

Previous reports have established that LuxS can regulate bacterial multicellular behavior such as biofilm formation [26], although mechanisms of the effect have not been fully elucidated. Some studies provided evidence that the effect of LuxS on biofilm formation is AI-2 dependent [26]. By contrast, some suggested a central metabolic role of LuxS in biofilm formation apart from the participation of AI-2 [27,28]. AI-2 is a byproduct of the LuxS-involving AMC, which is highly connected to the central metabolism and physiology of bacteria [29]. A *luxS* mutation would have pleiotropic effects and lead to complex metabolic change [27]. Therefore, the biological impacts of LuxS are very complex and should be carefully interpreted.

**Figure 1:**
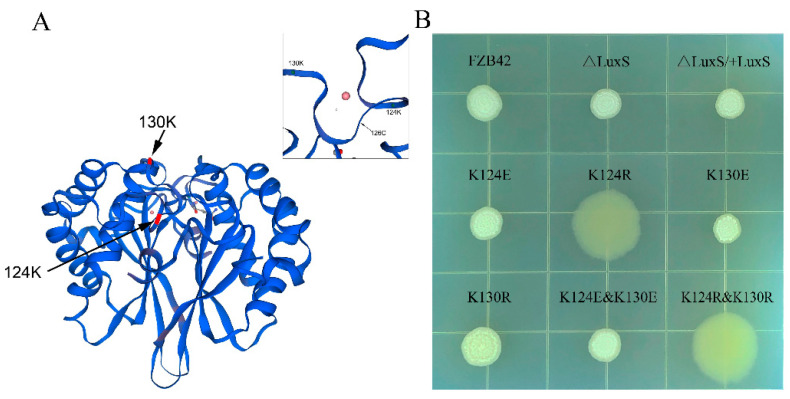
(**A**) Structure of LuxS of *B. velezensis* (FZB42) obtained by homology modeling with the web-based integrated service SWISS-MODEL [31]. (**B**) Colony morphology of FZB42 WT and its derived mutants grown on LBGM agar at 25 °C for 48 h.

The crystal structure of LuxS in *B. subtilis* has been determined [24,30]. The active sites of this LuxS include Cys-4, Glu-57, three Fe^2+^-chelating residues, His-54, His58, and Cys126 [10,31]. *B. velezensis* is phylogenetically similar to *B. subtilis*. The LuxS protein of *B. velezensis* (FZB42) is closely related to *B. subtilis* 168 with identity as high as 93.63%. The malonylation on the LuxS of FZB42 occurs on K124 and K130 (Figure 1A). It is intriguing to understand the roles of the two sites and their PTM on the functions of LuxS. Here, we constructed a series of LuxS mutants and performed measurements to learn the effects of the mutations on LuxS-related phenotypes and to explore the mechanisms underlying the function of LuxS.

## 2. Materials and Methods

Strains, plasmids and primers. Bacterial strains, primers, plasmids, and mutants used or constructed in this study are listed in Table 1.

Strain construction. The *luxS* sequence of FZB42 and its flanking regions were amplified with the primers FBO-445, FBO-453, and Pfu DNA polymerase. The PCR product was added with a -A tail using Taq DNA polymerase and then inserted into the commercial pMD-19 vector, resulting in the plasmid pMD-19-*luxS*. The primers FBO-449 and FBO-450 were used to inversely amplify pMD-19-*luxS* and then ligated with the *speR* gene, obtaining pFB106 (pMD-19-*luxS*-*speR*), which was transformed into FZB42 as described previously [32]. The *luxS* knockout strain FBS119 was obtained by screening the transformants.

The plasmid pFB01 [8] was double digested with AvaI and ClaI prior to use. The *luxS* was amplified with the primers FBO-465, FBO-495, and Pfu DNA polymerase. After double digestion by AvaI and ClaI, the *luxS* fragment was ligated with the digested pFB01, yielding pFB108, which was transformed into FBS119 similarly to above. The *luxS* complementation strain FBS121 was obtained by screening the transformants and DNA sequencing confirmation.

With pFB108 as the backbone, pFB145 was constructed to obtain the sequence for LuxS (K130E) mutation using the primers FBO-517 and FBO-518; pFB144 was constructed to obtain the sequence for LuxS (K130R) mutation using the primers FBO-516 and FBO-518; pFB109 was constructed to obtain the sequence for LuxS (K124R) mutation using the primers FBO-545 and FBO-547; pFB110 was constructed to obtain the sequence for LuxS (K124E) mutation using the primers FBO-546 and FBO-547; pFB146 was constructed to obtain the sequence for LuxS (K124R and K130R) mutation using the primers FBO-516 and FBO-518 and the pFB109 as the template; pFB147 was constructed to obtain the sequence for LuxS (K124E and K130E) mutation using the primers FBO-546 and FBO-547 and the pFB145 as the template.

The vectors pFB109, pFB110, pFB144, pFB145, pFB146, and pFB147 were transferred into FBS119 to obtain the corresponding mutation strains FBS122 (K124R), FBS123 (K124E), FBS124 (K130R), FBS125 (K130E), and the double mutation strains FBS126 (K124R and K130R) and FBS127 (K124E and K130E), respectively.

The primers FBO-1371 (with 6×His-tag sequence) and FBO-1372 were used to amplify the genomic DNA of FZB42 wild type, FBS122, FBS123, FBS124, and FBS125. The PCR products were then ligated with the StuI-digested vector pDG148-Stu as described previously [33]. The primer FBO-335 and FBO-336 were used to verify the transformants with recombinant vectors pFB487-pFB491, which were then transformed into *B. subtilis* 168 to obtain the LuxS over-expression strains FBS385–FBS389 (see Table 1).

Growth conditions. *Bacillus* and *Escherichia coli* strains were grown in LB at 37 °C, 200 rpm, except otherwise indicated. *V. harveyi* BB170 was grown in AB medium at 30 °C, 200 rpm. The AB medium contained 17.55 g NaCl, 12.3 g MgSO_4_ 7H_2_O, 2 g Casamino acids, 10 mL 1 M K_3_PO_4_ (pH7.0), 10 mL 0.1 M L-arginine, and 20 mL 50% glycerol. The pH was adjusted to 7.5 with 10 M KOH. The LBGM liquid medium contained 10 g NaCl, 5 g yeast extract, 10 g Tryptone, 1% glycerol, and 0.1 mM MnSO_4_ per liter. The solid LBGM medium contained 1.5% agar. Antibiotics were added where necessary at the following concentrations: ampicillin 100 mg mL^−1^, erythromycin 5 mg mL^−1^, kanamycin 5 mg mL^−1^, and spectinomycin 100 mg mL^−1^.

Biofilm formation. Biofilm formation at solid–air interface: 0.5 µL cultures of the FZB42 wild type and the mutants in a similar optical density were spotted onto a LBGM plate before being incubated at 25 °C for 48 h. Biofilm formation at liquid–gas interface: 0.5 µL cultures of the FZB42 wild type and the mutants in a similar optical density were added to a 24-well plate containing 1 mL liquid LBGM before being incubated at 25 °C for 60 h.

AI-2 bioassay. The FZB42 wild type and the mutants were shaken in LB overnight before being transferred (1%) into new LB for a continued incubation of 3 h. Then cell-free supernatants from the cultures were obtained by centrifugation. A single colony of *V. harveyi* (BB170) was transferred into AB medium and shaken at 30 °C, 200 rpm, till the optical density (OD_600_) reached around 0.3, when the cultures were centrifuged for bacterial pellets. Fresh AB media were added to the pellets adjusting the optical density to 0.5, and then supplemented with 10% of the *Bacillus* supernatants. Under the same incubation condition, the luminescence intensity of BB170 was monitored every half hour.

Spore quantification. The FZB42 wild type and the mutants were shaken overnight and then transferred into fresh LB medium (1:100 diluted). After incubation for 8, 16, and 24 h the cultures were heated at 85 °C for 10 min to kill the vegetative cells. After heat treatment, the cultures were five-time serially diluted with sterile water. Three microliters of diluted culture were spotted onto LB plates and incubated at 37 °C for 10 h before the calculation. Each treatment had three replicates.

Antibiotic production bioassay. *B. megaterium* and *Staphylococcus aureus* were cultured till OD_600_ = 1.0 before being 1:300 mixed with soft LB agar (50 °C). Then agar plates were prepared, and holes were punched. The pre-culture of the wild type and the mutants of FZB42 were transferred (1%) in fresh LB. After incubation for 3, 5, and 7 h, cell-free supernatants were prepared from the cultures. A total of 100 µL of the supernatants were added into the holes in the agar plates, which were then incubated at 37 °C for 12 h. Each treatment had three replicates.

Swarming motility. The strains were shaken overnight and then transferred (1%) into fresh LB medium for continued incubation till OD_600_ = 1.0. Ten microliters of the cultures were spotted in the center of LB plates containing 0.5% agar. Each treatment had three replicates. After incubation at 37 °C for 10 h with 70% humidity, the diameters of the colonies were measured.

Preparation of LuxS. The LuxS over-expression strains were cultured in LB at 37 °C and 200 rpm. When the OD_600_ reached 0.7, 1 mM IPTG was added into the culture to induce LuxS expression for 4 h. Then the cell pellets were collected by centrifugation at 8000 rpm. The pellets from each liter of cultures were washed and finally re-suspended with 100 mL PBS (pH 7.0). Subsequently, 2 mg mL^−1^ lysozyme, 2 mM EDTA, and 0.1 mM PMSF were added to the suspension and incubated at 37 °C for 30 min. Next, the pellets were subjected to ultrasonic treatment with a period of 6 s and an interval of 3 s. The nickel ion resin (Sangon Biotech, Shanghai, China) was used to purify the proteins. The ultrafiltration tubes (Millipore, Burlington, MA, USA) were used for the desalination of the protein solutions. The BCA kit (Generay, Shanghai, China) was used for protein quantification.

In vitro assay of LuxS activity. The 1 M HCl solution containing 10 mg mL^−1^ SAH was boiled at 100 °C for 20 min. After cooling, the pH of the solution was adjusted to 7.5. Sodium phosphate buffer (pH 7.5) was added to the solution to be a final concentration of 100 mM. The mixture was incubated at 37 °C for 15 min before the proteins were removed by centrifugation with ultrafiltration centrifuge tubes. The Ellman reagents were added to the mixture to determine the content of free sulfhydryl groups derived from the reaction product homocysteine. The reaction system containing 30 µL DTNB (10 mM), 1.5 mL sodium phosphate buffer (0.1 M, pH 8.0), 150 µL of the mixture above, and 1 mM EDTA was incubated at room temperature for 15 min before the absorbance at 412 nm was measured [34].

## 3. Results

### 3.1. Construction of Mutants

To understand the effects of the two malonylation sites of LuxS in *B. velezensis* FZB42, first, we knocked out *luxS* from the wild-type chromosome and then introduced a complementation at the *amyE* locus. Using the complementation construct, we replaced the two lysine residues (K124 and K130) with arginine or glutamic acid, obtaining the mutants FBS122 (K124R), FBS123 (K124E), FBS124 (K130R), FBS125 (K130E), respectively (Figure S1). Both arginine and glutamic acid are structurally similar to lysine. Like lysine, arginine is a basic polar amino acid; on the contrary, glutamic acid is an acidic polar amino acid. Based on the four mutants, we constructed double the mutants FBS126 (K124R and K130R) and FBS127 (K124E and K130E). DNA sequencing was performed, confirming that all mutants were correctly constructed.

### 3.2. K124 of LuxS Was Critical for Biofilm Formation of FZB42

Colony architecture is the form of biofilm that can be most easily detected. The strains were inoculated on LBGM agar plates and cultivated at 25 °C. After 48 h, most of the strains formed colonies with complex architecture, except the mutants FBS122 (K124R) and FBS126 (K124R and K130R), both of which formed flat, wet, and somewhat larger colonies without structure (Figure 1B). This indicated that the K124 was critical for biofilm formation, probably relating to its malonylation in cells.

**Figure 2:**
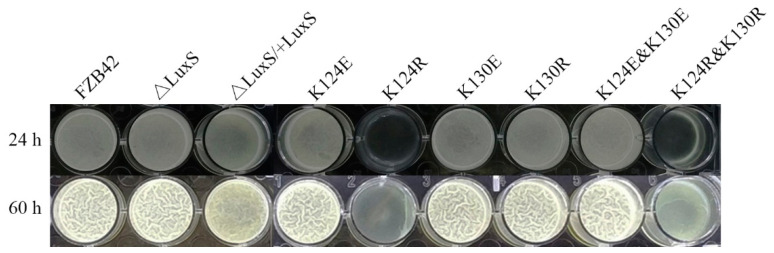
Pellicles formed by FZB42 WT and its mutants grown in LBGM for 24 h and 60 h.

To further check the effects of LuxS on biofilm formation, pellicles formed by the strains in liquid LBGM were also examined. After incubation for 24 h at 25 °C, all the strains except the mutant strains FBS122 (K124R) and FBS126 (K124R and K130R) formed thin pellicles (Figure 2). When cultured for 60 h, most strains formed pellicles with obvious wrinkles, whereas FBS122 (K124R) and FBS126 (K124R and K130R) had just started to form thin pellicles (Figure 2), indicating an obvious compromise in their biofilm formation ability. The scenario was consistent with their biofilm formation on agar.

Interestingly, the deletion mutant (Δ*luxS*) appeared to not be different from the FZB42 wild type in biofilm formation, whether on solid surfaces or liquid surfaces. The reason for this is still elusive but consistent with a previous report [28].

### 3.3. Mutations Compromised FZB42 Swarming and Sporulation

**Figure 3:**
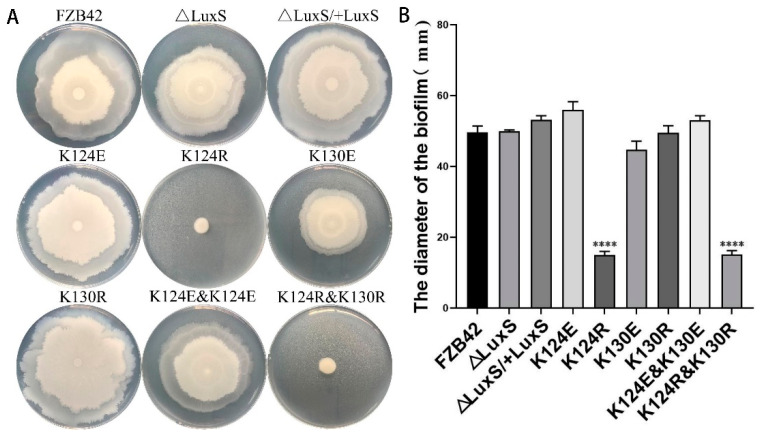
Swarming result of FZB42 WT and its derived mutants. The strains were grown on LB plates containing 0.5% agar at 37 °C for 10 h. (**A**) A representative set of plates showing the colonies after incubation. (**B**) Statistics of the diameters of colonies formed by the strains. ****(*p* < 0.0001).

The biofilm formation of *Bacillus* species is closely related to bacterial motility and sporulation. The effect of the mutations on biofilm formation led us to consider if they could also affect the features such as motility and sporulation. A swarming assay was performed to check the motility abilities of the strains. It was shown that, similar to biofilm formation, FBS122 (K124R) and FBS126 (K124R and K130R) completely abolished swarming ability, while the other mutants, including the ∆*luxS* mutant, revealed an intact swarming proficiency as the wild type (Figure 3).

**Figure 4:**
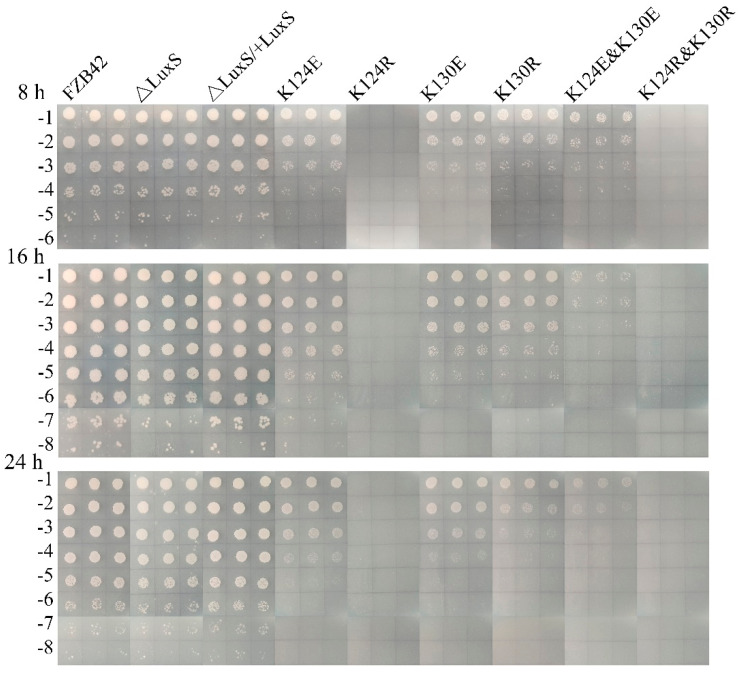
Quantification of sporulation in FZB42 wild type and its mutants. The strains were grown in LB for the time indicated before being subjected to heat treatment. The culture was serially diluted five times. Three microliter aliquots of the cultures were spotted onto LB agar and incubated at 37 °C for 10 h.

Endospores of the strains were quantified after 8, 16, and 24 h. The sporulation rate showed a similar pattern at the three-time points. While sporulation was not affected by the deletion of *luxS* (∆*luxS*), all lysine substitution mutants showed a pronounced sporulation defect (Figure 4). FBS122 (K124R) and FBS126 (K124R and K130R) failed entirely to sporulate. FBS123 (K124E) and FBS125 (K130E) displayed a reduced sporulation rate, and FBS127 (K124E and K130E) had an even lower rate, suggesting a synergistic effect of double mutations. FBS124 (K130R) had a roughly five times lower sporulation rate than FBS125 (K130E), resembling the effect of the two K124 mutants. Therefore, we conclude that both amino acids, K124 and K130, are essential for the normal sporulation of FZB42.

### 3.4. K124R and K130R Induced AI-2 Production

The involvement of LuxS in the biofilm formation of some bacteria is thought to be implicated with AI-2 generation. To understand this mechanism in more detail, we used *V. harveyi* (BB170) (*luxN::Tn5*) to check the effect of the LuxS mutation on the production of AI-2. BB170 can be activated by AI-2 molecules in the environment to emit luminescence, thereby indicating the presence of AI-2 molecules.

**Figure 5:**
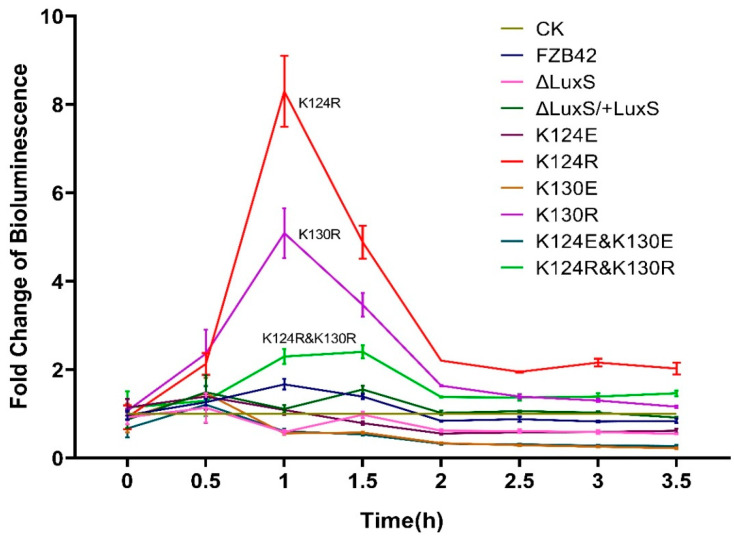
Induction of bioluminescence in *V. harveyi* (BB170) by cell-free supernatants of FZB42 WT and its LuxS mutants. At time zero, the supernatants were added to BB170 cultures at a final concentration of 10% (*vol/vol*), and light production was recorded. In the CK, only LB was added. Three replicates were performed for each treatment.

There was no obvious difference between the control containing only medium with the supernatants from most strains, except FBS122 (K124R), FBS124 (K130R), and FBS126 (K124R and K130R), which clearly elevated luminescence of BB170 at 0.5–2.0 h after supernatant addition (Figure 5). The highest promotion of luminescence occurred with the K124R supernatant, leading to an eight-fold change at the first hour. The supernatant of FBS124 (K130R) increased luminescence intensity but not as high as FBS122 (K124R). The double mutant FBS126 (K124R and K130R) also had an induced luminescence intensity, but surprisingly, it was lower than FBS122 (K124R) and FBS124 (K130R) for an unknown reason. The enhancement of BB170 luminescence intensity informed that the three mutants produced more AI-2 than the others.

### 3.5. Mutations Enhanced Antibiotic Production

**Figure 6:**
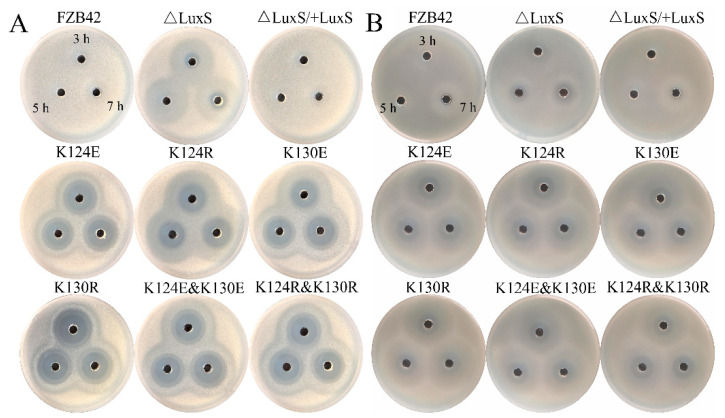
Antagonistic activities of FZB42 WT and its derived mutants against the indicator strains *B. megaterium* (**A**) and *S. aureus* ATCC 9144 (**B**). The cell-free supernatants of the strains prepared from the 3rd, 5th, and 7th hour were filled into the holes. Inhibition zones were examined after overnight incubation.

*B. velezensis* (FZB42) can produce multiple antibiotics, including the polyketide difficidin and the dipeptide bacilysin, both of which have strong activity against a broad spectrum of bacteria [6,35,36,37]. They may affect *V. harveyi* (BB170) growth and then its bioluminescence generation. We used *B. megaterium* and *S. aureus* ATCC9144 as indicator strains [38,39] to evaluate the effects of the mutations on the production of the two antibiotics. Effects of the culture supernatants of the strains incubated for 3, 5, and 7 h were compared. Whenever the supernatants were collected, all the substitution mutations showed an increased inhibitory zone against *B. megaterium*, indicating enhanced production of the antibiotics (Figure 6A). The two double mutants displayed an activity as strong as their corresponding single mutants. The ∆*luxS* mutant displayed only a slightly increased antibiotic production, less than the other substitution mutants. The complementation of *luxS* at the *amyE* locus completely recovered the wild-type phenotype. Effects of the mutations on antibiotic production, as indicated by *S. aureus* (Figure 6B), were highly like the effect on *B. megaterium*.

### 3.6. K130 Mutations Affected in vitro Enzymatic Activity of LuxS

**Figure 7:**
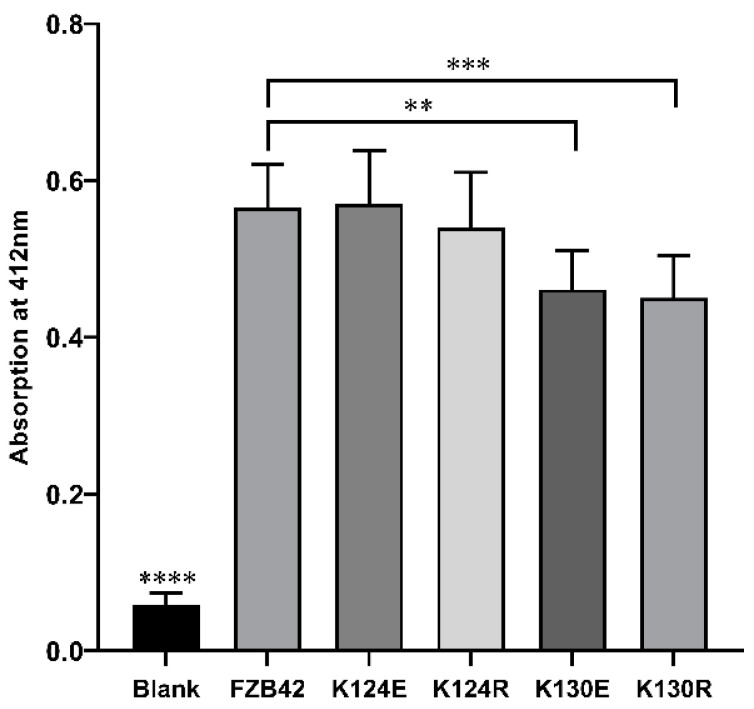
In vitro enzymatic activities of the intact LuxS of FZB42 and its derivates with mutations. ** (*p* = 0.0023), *** (*p* = 0.0008), **** (*p* < 0.0001).

To figure out if the mutations affect the catalytic activity of LuxS directly, we expressed the LuxS proteins in *B. subtilis* 168, then isolated and purified them to perform in vitro assays. The results revealed that all the purified proteins could catalyze the reaction (SRH ⇋ L-homocysteine + DPD) in vitro (Figure 7). LuxS prepared from FBS122 (K124R), and FBS123 (K124E) showed no significant difference from the wild type LuxS. The activities of the two K130 mutated LuxS (K130R and K130E) were similar to each other but lower than the wild type and the two K124 mutated proteins. It seems that the effect pattern of the mutations on enzymatic activities did not fit, at least in vitro, with their effect pattern on the AI-2 production, as indicated by the bioluminescence assay. The reason for this inconsistencey is yet unknown.

### 3.7. K130 Is Highly Conserved in Bacillus Species

**Figure 8:**
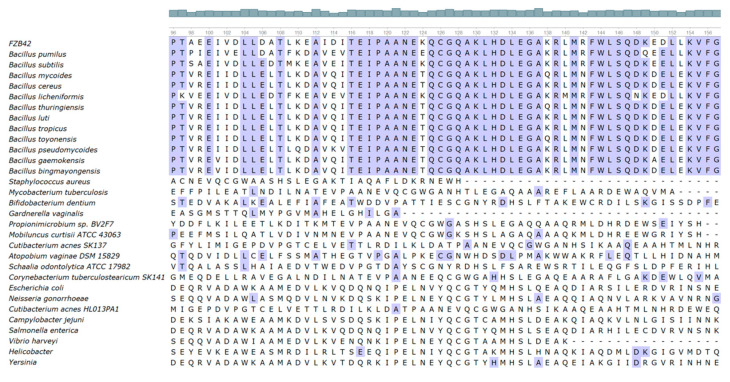
Alignment of the amino acid sequences of LuxS from different bacteria.

The active sites of *B. subtilis* LuxS contain Cys84, Glu57, and the Fe^2+^-chelating His54, His58, and Cys126 residues [10]. To know the degree of conservation of K124 and K130, we retrieved LuxS homologous sequences of 30 strains from NCBI, including 12 *Bacillus* species, 10 *Actinomycete* species, and 8 Gram-negative *Proteobacteria* species. The sequence alignment revealed that amino acids in the active site region of LuxS in various *Bacillus* species shared a high degree of identity, but not in the non-*Bacillus* strains. In *Bacillus*, K130 is highly conserved, but K124 is not conserved (Figure 8).

## 4. Discussion

In this work, two lysine sites that can be malonylated, K124 and K130, were found to regulate the effects of LuxS on different phenotypic features of *B. velezensis* (FZB42), such as biofilm formation, motility, sporulation, and antibiotic production. Effects of the two sites on AI-2 production and the in vitro enzyme activity of LuxS were also examined in order to explore the underlying mechanisms for LuxS functions. To the best of our knowledge, this was the first report investigating the roles of LuxS in *B. velezensis*, the species widely used as microbial fertilizer agents.

According to our previous study, protein malonylation highly occurs on the nonribosomal peptide synthetase (NRPS) enzyme complexes of FZB42 [1], indicating their possible impacts on the production of antimicrobial secondary metabolites. Furthermore, there are also two malonylation sites detected on LuxS, which are reported to affect biofilm formation and motility in some bacteria [26,28]. In *Bacilli*, biofilm and motility, together with sporulation and antibiotic synthesis, are regulated by a network with some shared regulators [40]. Given that LuxS is a relatively small protein (17.6 Dalton) with only two malonylation sites and its gene *luxS* being monocistronic, it is an ideal target to elucidate the effects of malonylation sites on protein activities.

The LuxS mutants of FZB42 investigated here showed evident differences in their effects on biofilm formation. The mutation K124R and the double mutation (K124R and K130R) impaired biofilms most severely, whereas the other mutants did not result in significant differences from the wild type, which is characterized by forming robust biofilms with complex architecture. Furthermore, the Δ*luxS* mutant could also form normal biofilms, which seems to disagree with some reports that LuxS participates in biofilm formation [26,41]. However, this result is not unique. For example, Duanis-Assaf et al. also found that their *B. subtilis* wild type and Δ*luxS* cells displayed similar pellicle formation in LBGM [28]. Ju et al. even found that the loss of *luxS* promoted biofilm formation by *Salmonella serovar* Dublin and the expression of biofilm-related genes [42]. The reason for these contradictions remains intangible since mechanisms by which *luxS* affects biofilm formation are still unclear. We assume that these “contradictive” findings are linked with the complexity of the regulatory networks of biofilm formation.

Some studies showed that the signaling molecule AI-2, the byproduct of LuxS activity, is involved in the effect on biofilm formation. For example, the AI-2 molecule has been shown to be able to up-regulate the expression of the EPS biosynthesis genes in *V. cholerae* [43]. In addition, the addition of synthesized DPD could partially restore the bundling phenotype of the Δ*luxS* mutant of *B. subtilis* [28], while blocking the AI-2 pathway using an AI-2 analog impaired biofilm formation by *B. subtilis* [44]. To figure out the relationship between the effects of our LuxS mutation on biofilm with AI-2, we examined their effects on the production of AI-2. The bioluminescence intensity of BB170 supplemented with supernatants of FBS123 (K124E), FBS125 (K130E), and FBS127 (K124E and K160E) was generally in the same range as in the Δ*luxS* mutant and the wild type. Since all the mutants produced more antibiotics (see the results above and discussion below), which may inhibit their growth, we actually cannot draw a conclusion about how the deletion of *luxS* or replacing lysine with glutamic acid affects AI-2 production. In contrast, when lysine was mutated to arginine (K124R, K130R, and K124R and K160R), BB170 luminescence intensity was much higher than that with the wild type supernatant, suggesting that the production of AI-2 was enhanced by these mutations despite the increased antibiotic inhibition. Consequently, it seems that a high level of AI-2 does not induce but weaken biofilm formation.

In fact, the roles of AI-2 in biofilm formation are also elusive. A study has shown that adding exogenous AI-2 could not restore the biofilm formation of *luxS*-deficient strains [27]. Moreover, the addition of exogenous AI-2 had an inhibitory effect on biofilm formation by *B. cereus* [45]. Lebeer et al. have argued [27] that the central metabolic role that LuxS also plays has a strong effect on biofilm formation. Deletion of *luxS* can interfere with primary bacterial metabolism and thus change their nutrition requirements. Taken together, it is very likely that the effect of LuxS on biofilm formation only partly acts through AI-2. We further assume that there might be a back-up pathway serving to overcome the defect of the absence of *luxS*, which is necessary for detoxication of SRH accumulation and recycling of homocysteine. Due to the importance of AMC in the central metabolism, the absence or decreased activity of *luxS* may activate the back-up system, continuing the cycle and producing enough AI-2 as normal.

A recent report provides some clues for the mechanism of AI-2 involved in biofilm formation [12]. For a long time, people did not know what the receptors of AI-2 were in many bacteria that lack LuxP and LsrB [46,47]. Zhang et al. recently demonstrated that KinD of *B. subtilis* is a novel type of AI-2 receptor [12]. AI-2 can enhance the kinase activity of KinD by binding to its dCACHE domain. Obviously, through the well-known phosphorelay [39,48,49], signaling from KinD to Spo0A may help to explain the mechanisms of AI-2/LuxS involved in biofilm formation. It is possible that, via triggering of the kinase activity of KinD, overproduced AI-2 results in a high level of Spo0A~P, which then inhibits biofilm formation.

We also tested the motility and sporulation of these mutants. Their effects on swarming were found to be very similar to those on biofilm formation. Only the mutants FBS122 (K124R) and FBS126 (K124R and K130R) had significantly reduced bacterial swarming ability, while the other strains, including the Δ*luxS* mutant, resembled the wild type strain. Therefore, it is likely that these mutations affect swarming through pathways connected with biofilm formation. Their effect on sporulation, on the one hand, resembled their effects on biofilm formation and swarming. FBS122 (K124R) and FBS126 (K124R and K130R) were not heat resistant at all. Microscopic observation confirmed that they did fail to produce endospores (data not shown) rather than forming endospores defected in heat resistance, indicating that their sporulation ability was entirely abolished. On the other hand, FBS123 (K124E), FBS125 (K130E), and FBS127 (K124E and K130E) exhibited defected, but not completely deficient, sporulation ability. FBS127 exhibited a more serious defection than FBS123 and FBS125, indicating a synergistic effect of the two K to R substitutions. All the mutants were altered in sporulation, implying that *luxS* was deeply embedded in the sporulation regulatory network. Surprisingly, the Δ*luxS* mutant still presented a wild-type phenotype. While the reason for this phenomenon is still obscure, this result is not a solitary finding but in agreement with a previous report [26]. As we assumed in the case of biofilm formation, in sporulation, the lost roles of *luxS* might be compensated by the back-up circuit. By contrast, the amino acid substitution mutations did not activate the back-up circuit since the LuxS molecules were still present despite possible defects.

FZB42 is a potent producer of multiple antibiotics, whose production is governed by many regulators that are also acting in biofilm formation, swarming, and sporulation [6,38,50,51,52,53,54]. We were, therefore, curious about the effects of these mutations on antibiotic production as well. Some of the antibiotics, mainly difficidin and bacilysin, have strong inhibitory action against many bacteria [6,51]. Therefore, it is also necessary to evaluate the influences of the supernatants containing both AI-2 and possible antibiotics. Most of the antibiotics are produced since the late-exponential phase of FZB42 growth (usually after 12 h). We performed a pilot experiment using the FZB42 wild type (Figure S2). It was shown that the supernatants collected from cultures of longer incubation (>6 h) resulted in strongly decreased luminescence intensity, indicating an inhibitory effect of the antibiotics on BB170 growth. To avoid this inhibitory effect, we used the supernatants from the 3 h culture, which had little effect on BB170 growth but could cause obvious QS response for the AI-2 production assay.

Neither the wild type nor the complementation strain could significantly inhibit *B. megaterium*. This is probably because the supernatants were collected from cultures prior to the stage of antibiotic production. *B. megaterium* appeared more sensitive than *S. aureus* ATCC9144 to the antibiotics. Concerning the *B. megaterium* results, if the most transparent zones near the holes are regarded as the effect of one antibiotic, it can be noticed that the production of this antibiotic is enhanced in all mutants except Δ*luxS*. This seems to some extent similar to their effects on sporulation. Although it is hard to distinguish the antibiotics only from inhibitory zones, the similar effect patterns seem consistent with our assumption that the effects of LuxS on these features act through some common regulators or pathways.

Although K124 is located closer to the active center of LuxS than K130 (Figure 1A), the in vitro enzyme activity assay demonstrated that the mutations K124R and K124E had no significant effect on LuxS catalytic activity, while the mutations K130R and K130E significantly decreased LuxS activity. This result may be consistent with the conservation of K130, suggesting an important role of this lysine residue. The effect patterns of the mutations on the in vitro enzyme activity of LuxS are not similar to any effect patterns above, which, however, seem to again support the hypothesis that there is a back-up circuit for LuxS regulation.

In summary, we demonstrated that two lysine residues that can be malonylated, are important for LuxS biological roles in governing biofilm formation, swarming, sporulation, and antibiotic production. In the future, in-depth studies can be performed to uncover the molecular mechanisms underlying the effects. In addition, applying omics technologies to search for regulatory factors that were affected by specific amino acid substitutions may offer new clues for unveiling functions of LuxS in more detail.

## Figures and Tables

**Table 1:** Strains, plasmids, and primers used in this study.

Materials	Description or Sequence (5′–3′)	Source
Strain		
FZB42	*Bacillus velezensis* FZB42 wild type	Lab stock
FBS119	FZB42, *luxS*::*speR*	This study
FBS121	FZB42, *amyE*::*luxS* + *emR*, *luxS*::*speR*	This study
FBS122	FZB42, *amyE*::*luxS* (K124R) + *emR*, *luxS*::*speR*	This study
FBS123	FZB42, *amyE*::*luxS* (K124E) + *emR*, *luxS*::*speR*	This study
FBS124	FZB42, *amyE*::*luxS* (K130R) + *emR*, *luxS*::*speR*	This study
FBS125	FZB42, *amyE*::*luxS* (K130E) + *emR*, *luxS*::*speR*	This study
FBS126	FZB42, *amyE*::*luxS* (K124R,K130R) + *emR*, *luxS*::*speR*	This study
FBS127	FZB42, *amyE*::*luxS* (K124E,K130E) + *emR*, *luxS*::*speR*	This study
*V. harveyi* BB170	*luxN::Tn5*	BNCC
*B. megaterium*		Lab stock
*S. aureus* ATCC9144	bacilysin sensitive	ATCC
*B. subtilis* 168	*trpC2*	BGSC
FBS385	*B. subtilis* 168 with pDG148-Stu-*luxS* (K130E)	This study
FBS386	*B. subtilis* 168 with pDG148-Stu-*luxS* (K130R)	This study
FBS387	*B. subtilis* 168 with pDG148-Stu-*luxS*	This study
FBS388	*B. subtilis* 168 with pDG148-Stu-*luxS* (K124E)	This study
FBS389	*B. subtilis* 168 with pDG148-Stu-*luxS* (K124R)	This study
Plasmids		
pFB01	amyE::*gfp* + *emR* (Amp^r^ Em^r^)	[8]
pMD-19	Commercial T-Vector (Amp^r^)	Takara
pFB68	pDG148-Stu (Amp^r^ Km^r^)	BGSC
pFB106	pMD-19-*luxS*-speR (Amp^r^ Spc^r^)	This study
pFB108	pFB01-*luxS* (Amp^r^ Em^r^)	This study
pFB109	pFB01-*luxS*(K124R) (Amp^r^ Em^r^)	This study
pFB110	pFB01-*luxS*(K124E) (Amp^r^ Em^r^)	This study
pFB144	pFB01-*luxS*(K130R) (Amp^r^ Em^r^)	This study
pFB145	pFB01-*luxS*(K130E) (Amp^r^ Em^r^)	This study
pFB487	pDG148-Stu-*luxS*-K130E (Amp^r^ Km^r^)	This study
pFB488	pDG148-Stu-*luxS*-K130R (Amp^r^ Km^r^)	This study
pFB489	pDG148-Stu-*luxS* (Amp^r^ Km^r^)	This study
pFB490	pDG148-Stu-*luxS*-K124E (Amp^r^ Km^r^)	This study
pFB491	pDG148-Stu-*luxS*-K124R (Amp^r^ Km^r^)	This study
Primers		
FBO-335	ATAATCCACAGCAGGTA	
FBO-336	TTGAACAATCACGAAAC	
FBO-445	ATACCAAACATCTAAATTCCCGG	
FBO-449	CGATCACTTCGACATCATAGATA	
FBO-450	GCAGAAGCGAATGTCAAACTTAT	
FBO-453	TTGTTCTGCGCTCTCATTGC	
FBO-465	ATAATCGATCTTCCGCCACAATTCTTA (ClaI restriction site)	
FBO-495	ATACTCGAGAGCATACCGCACATACCT (AvaI restriction site)	
FBO-516	ATCATGAAGTCTCGCCTGGCC	
FBO-517	ATCATGAAGTTCCGCCTGGCC	
FBO-518	TTAGAAGGCGCGAAACGTCTGAT	
FBO-545	GCGGCCAACGAAAGACAGTGC	
FBO-546	GCGGCCAACGAAGAACAGTGC	
FBO-547	CGGAATCTCCGTAATATCGAT	
FBO-1371	AAGGAGGAAGCAGGTATGCATCACCATCACCATCACGGATCAATGCCTTCATAGAAAGTTTTGAG (His-tag)	
FBO-1372	GACACGCACGAGGTTTATCCGAACACTTTCAGCAAATC	

## Supplementary Materials

The following are available online at https://www.mdpi.com/article/10.3390/microorganisms9061338/s1, Figure S1: Schematic diagram of the construction of the *luxS*-related mutants, Figure S2: Induction of bioluminescence in *V. harveyi* BB170 by cell-free supernatants of FZB42 WT collected at different time.
