# Supplementary material for: Two Lysine Sites That Can Be Malonylated Are Important for LuxS Regulatory Roles in Bacillus velezensis
Source: Microorganisms. 2021 Jun 21;9(6):1338. doi: 10.3390/microorganisms9061338 (PMC8233902; doi:10.3390/microorganisms9061338)
Supplement: Supplementary file 1 [file microorganisms-09-01338-s001.zip › microorganisms-1183903-supplementary.pdf]

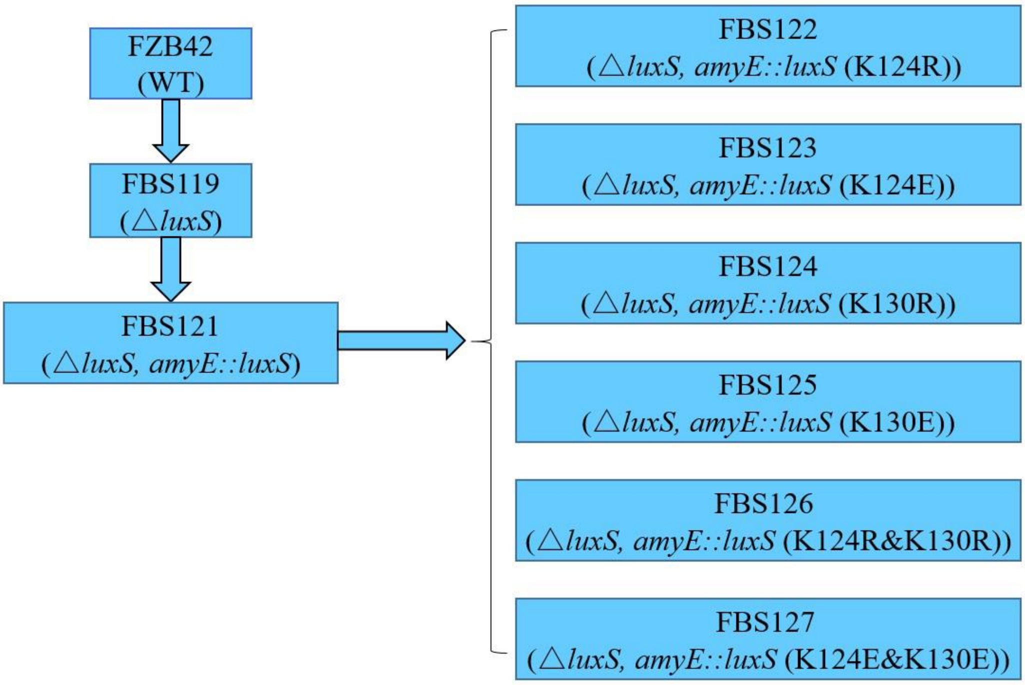

Figure S1. Schematic diagram of the construction of the *luxS*-related mutants.

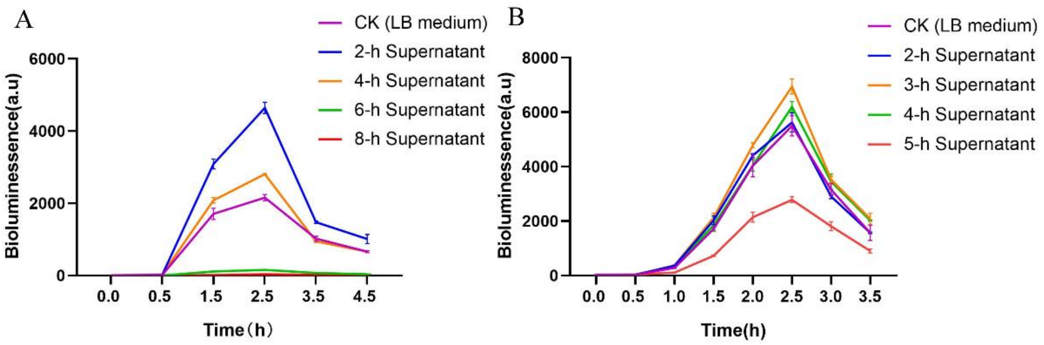

Figure S2. Induction of bioluminescence in *V. harveyi* BB170 by cell-free supernatants of FZB42 WT collected at different time.
